# Experimental cross-species infection of donkeys with equine hepacivirus and analysis of host immune signatures

**DOI:** 10.1186/s42522-022-00065-y

**Published:** 2022-05-09

**Authors:** André Gömer, Christina Puff, Birthe Reinecke, Stephanie Bracht, Maria Conze, Wolfgang Baumgärtner, Jörg Steinmann, Karsten Feige, Jessika M. V. Cavalleri, Eike Steinmann, Daniel Todt

**Affiliations:** 1 Department of Molecular and Medical Virology, Ruhr University Bochum, Bochum, Germany; 2 Institute of Virology, University of Veterinary Medicine Hannover, Foundation, Hannover, Germany; 3 Department of Pathology, University of Veterinary Medicine Hannover, Foundation, Hannover, Germany; 4 Institute of Experimental Virology, TWINCORE Center for Experimental and Clinical Infection Research, Hannover, Germany; 5 Clinic for Horses, University of Veterinary Medicine Hannover, Foundation, Hannover, Germany; 6 Institute of Medical Microbiology, University of Hospital Essen, University of Duisburg-Essen, Essen, Germany; 7 Institute of Clinical Hygiene, Medical Microbiology and Infectiology, General Hospital Nürnberg, Paracelsus Medical University, Nürnberg, Germany; 8 Clinical Unit of Equine Internal Medicine, Department for Companion Animals and Horses, University of Veterinary Medicine Vienna (Vetmeduni), Vienna, Austria; 9 European Virus Bioinformatics Center (EVBC), Jena, Germany

## Abstract

**Background:**

The Equine Hepacivirus (EqHV) is an equine-specific and liver-tropic virus belonging to the diverse genus of Hepaciviruses. It was recently found in a large donkey (*Equus asinus*) cohort with a similar seroprevalence (30%), but lower rate of RNA-positive animals (0.3%) compared to horses. These rare infection events indicate either a lack of adaptation to the new host or a predominantly acute course of infection.

**Methods:**

In order to analyze the susceptibility and the course of EqHV infection in donkeys, we inoculated two adult female donkeys and one control horse intravenously with purified EqHV from a naturally infected horse. Liver biopsies were taken before and after inoculation to study changes in the transcriptome.

**Results:**

Infection kinetics were similar between the equids. All animals were EqHV PCR-positive from day three. EqHV RNA-levels declined when the animals seroconverted and both donkeys cleared the virus from the blood by week 12. Infection did not have an impact on the clinical findings and no significant histopathological differences were seen. Blood biochemistry revealed a mild increase in GLDH at the time of seroconversion in horses, which was less pronounced in donkeys. Transcriptomic analysis revealed a distinct set of differentially expressed genes, including viral host factors and immune genes.

**Conclusion:**

To summarize, our findings indicate that donkeys are a natural host of EqHV, due to the almost identical infection kinetics. The different immune responses do however suggest different mechanisms in reacting to hepaciviral infections.

**Supplementary Information:**

The online version contains supplementary material available at 10.1186/s42522-022-00065-y.

## Introduction

The genus Hepacivirus comprises numerous viruses, each with a strict species tropism infecting a specific host, including mammals, reptiles, and birds [1]. The hepatitis C virus (HCV) exclusively infects human hepatocytes, amounting to more than 70 million people worldwide [2]. A cross-species transmission from humans to their closest relative, the chimpanzee, is however only achievable via experimental infection [3, 4]. The Equine Hepacivirus (EqHV), originally found to infect horses (*Equus caballus*) [5, 6], was recently also found in donkeys (*Equus asinus*) in a diverse cohort which sampled sera between 1974 to 2016 from Germany, Spain, Bulgaria, Italy, France, Mexico, and Bulgaria [7]. EqHV viral loads were comparable in both species, but slightly elevated levels of serum liver enzymes were only apparent in horses [7–9]. While the seroprevalence in donkeys (31.5%; 278/882) was comparable to that of horses (30–40% in North America or Europe), viral RNA has only been detected in 0.3% (3/882) of the animals, which is lower to what has been observed for horses (2–8%) [7, 10–14]. The absence of chronically infected donkeys and the lower rate of EqHV RNA-positive donkeys suggests that the disease might rather be acute instead of persistent, leaving speculations as to whether donkeys might be more resistant to EqHV than horses [7].

Both species belong to the order of Perissodactyla which includes three families: Tapiridae, Rhinocerotidae and Equidae. The rapidly evolving family of Equidae separated approximately 4–4.5 million years before present [15, 16] and consists of a single genus, *Equus* [17]. This genus comprises horses (*Equus ferus*), donkeys (*Equus asinus*) and zebras (*Equus hippotigris*) which are all able to hybridize. Hybrids are almost always sterile, due to the high chromosomal plasticity within the genus (*n* = 16–33 chromosomes), which hinders, but not prohibits, fertility among hybrids [18, 19]. Hybridization of a horse (*n* = 32) and a donkey (*n* = 31) result in either a hinny (male horse x female donkey) or mule (female horse x male donkey), showing that the two species are closely related. Moreover, donkeys were shown to share a wide variety of diseases with horses, including viral infections such as Equine Infectious Anemia (EIA), African Horse Sickness and West Nile Fever (reviewed in [20]). However, these were in many cases reported to have a milder course of disease in donkeys than in horses, indicating a higher resistance in donkeys, probably caused by a different immune response against the viruses [20].

In order to analyze susceptibility, course of infection and immune response of donkeys to EqHV infection, we inoculated two adult female donkeys and one control horse intravenously with purified EqHV from a naturally infected horse. Animals underwent daily clinical examinations and blood was drawn at weekly intervals for 15 weeks. Hematological data, plasma GLDH, GGT, AST and fibrinogen were evaluated. In serum, anti-NS3-EqHV antibodies were analyzed by luciferase immunoprecipitation system (LIPS) and viral load was quantified using real-time PCR. Liver biopsies were taken before and after inoculation to study changes in the transcriptome.

## Materials and methods

### Animals & ethical statement

In this study we inoculated two donkeys, age 5 and 25 years, and one Icelandic horse, age 23 years, with each 10 mL ultra-centrifugation purified EqHV-positive serum each (dissolved in 0.9% sterile NaCl, 8.4 × 10^5^ EqHV RNA copies per mL) from a previously characterized chronically infected horse (EqHV sequence H12 in [21], NCBI accession number KP739812). Animal experiments were first examined by the animal welfare representatives of the University of Hannover Foundation, and then approved by the Lower Saxony’s official authorities (LAVES 33.19–42,502–04-16/2143).

### Viral RNA quantification

RNA from plasma was isolated using the High-Pure Viral RNA Kit (Roche, Mannheim, Germany) according to the manufacturer’s manual and random hexamer primer were used to generate cDNA (Prime Script RT Master Mix Kit, Takara). For quantification of EqHV RNA, SYBR Premix Ex Taq II kit (Takara) with previously published primers targeting the 5′ untranslated region (5’UTR) were used [12]. A standard curve for copy number calculation was implemented by serial dilution of in vitro transcribed RNA containing the 5’UTR of EqHV isolate NPHV-NZP-1 (JQ434001).

### Lips

The Luciferase immunoprecipitation system (LIPS) was performed as previously described [12]. Serum samples were diluted 1:10 in buffer A (50 mN Tris [pH 7.5], 100 mM NaCl, 5 mM MGCL2, and 1% Triton X-100). 1 × 10^7^ relative light units (RLU) of a Renilla-luciferase-NS3-antigen fusion (RLUC-NS3) was mixed into 40 μL buffer A and subsequently added to 10 μL of diluted sera for 1 h shaking at room temperature. For antigen precipitation: 30% Ultralink protein A/G beads (Pierce Biotechnology, Rockford, IL) were added to a 96-well filter HTS plate (Millipore, Bedford, MA), before 100 μL of the RLUC-NS3 antigen serum mixture was added for 1 h shaking at RT. Afterwards the filter plate was washed on a vacuum plate washer. Antigen-precipitation was quantified by adding 100 μL coelenterazine (p.j.k. GmbH, Kleinblittersdorf, Germany) on a Berthold LB960 centro XS3 plate luminometer (Berthold, Freiburg). The threshold for NS3-antibody positive samples was set at three standard deviations above the average of the negative controls.

### Histology and immunohistochemistry

Liver biopsies were fixed in 10% neutrally buffered formalin, routinely embedded in paraffin wax, sectioned at 2–3 μm and stained with hematoxylin and eosin. Periportal inflammatory cell infiltrates were characterized by immunohistochemistry using the ABC method with 3,3′-diaminobenzidine as a chromogen. Primary antibodies were directed against CD3 (1:1000, rabbit polyclonal, DakoCytomation), Pax-5 (1:100, mouse monoclonal, clone 24/Pax-5, BD Transduction) and myeloid/histiocyte antigen (1:500, mouse monoclonal, clone MAC387, DakoCytomation) as previously described [22, 23]. Heat-induced antigen retrieval by boiling in citrate buffer for 20 min was performed for all antibodies. Slides were analyzed by counting the number of periportally localized immunopositive cells. The obtained result was divided by the total number of inflammatory cells (sum of immunolabelled cells in all three staining) to gain the relative amount of each inflammatory cell type.

### Fluorescence in situ-hybridization

Fluorescence in situ-hybridization (FISH) was used to detect EqHV within formalin-fixed paraffin-embedded sections of the liver biopsies. FISH was conducted using an isolate specific RNA probe mix targeting the EqHV NS3 domain and based on NS3 nucleotide sequence information of the inoculated EqHV (Affymetrix-Panomics, Santa Clara, CA, USA). FISH was performed according to manufacturer’s instructions as previously described (QuantiGene ViewRNA ISH Tissue 1-Plex Assay Kit; QuantiGene ViewRNA Chromogenic Signal Amplification Kit; Affymetrix, Santa Clara, CA, USA) [24]. Briefly, after deparaffinization, slides were incubated in pretreatment solution for 20 min at 85–90 °C and digested by protease QF for 10 min followed by hybridization for 6 h. After preamplification and amplification steps, slides were incubated with Fast Red substrate and counterstained with Mayer’s hemalaun (Carl Roth GmbH, Karlsruhe, Germany). Images were obtained with an inverted fluorescence microscope (Olympus IX-70; Olympus Life Science Europe GmbH, Hamburg, Germany). Analysis was performed by determination of the percentage of positive hepatocytes in five randomly selected fields per sample at a 20x magnification. Additionally, a negative control consisting of a non-probe incubation was performed for each sample and an EqHV-positive horse served as a positive control (Fig. S1).

### RNA-seq analysis

RNA from liver biopsies was isolated using the Machery Nagel RNA extraction kit according to manufacturer’s instructions. Mechanical homogenization was done in the Machery Nagel RNA extraction buffer using an IKA ULTRA-TURRAX homogenizer. The quality of RNA was accessed via gel electrophoresis and an Agilent Bioanalyzer Nano Chip. A poly-A enrichment library preparation was done using NuGEN Universal Plus mRNA-Seq-Kit with 1000 ng RNA input. Paired end sequencing was done at the sequencing facility of the University-hospital Essen using an Illumina Hiseq2500 platform. Demultiplexing of reads was done with CASAVA 1.8.2. Raw reads were quality trimmed and mapped to the reference genome for horses (EquCap3.0) and donkeys (ASM303372v1), respectively, using CLC Genomics Workbench 21.0.3. Data was visualized in R using the following packages: tidyverse, ggplot2, ggpubr, cowplot, ggally, DOSE, GO-plot, clusterprofiler.

## Results

### Donkeys are susceptible to experimental inoculation of EqHV with a similar course of infection compared to horses

**Fig. 1:**
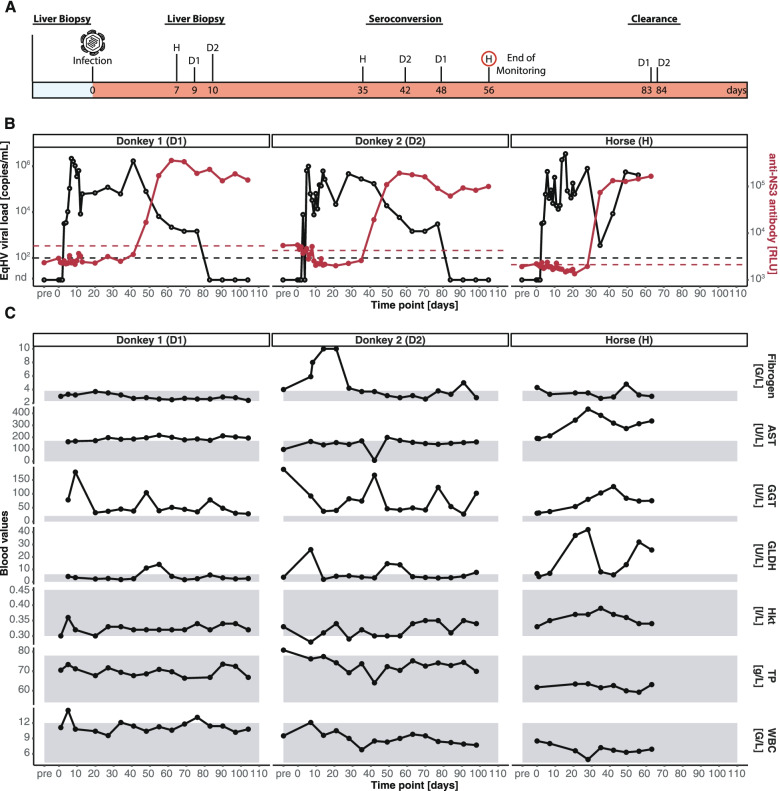
Study design and course of disease. **A** Study design: Two donkeys and one horse were intravenously inoculated with purified EqHV-positive serum. **B** EqHV genome equivalents (black line) and anti-NS3 antibody course (red line) during the observation period. **C** Plasma levels of fibrinogen (G/L), aspartate transaminase (AST, U/L), gamma-glutamyl transferase (GGT, U/L), glutamatdehydrogenase (GLDH, U/L), hematocrit (Hkt, I/L), total protein (TP, g/L), and white blood cell counts (WBC, G/L). Reference intervals are indicated in grey

To evaluate whether EqHV can be experimentally transmitted to donkeys and to which extent donkeys are susceptible, we inoculated two naïve female donkeys and one naïve female horse with the virus (Fig. 1A). To this purpose, we purified EqHV-positive serum from a horse via ultra-centrifugation and subsequently dissolved the corresponding fraction in 0.9% sterile sodium chloride. Then, we infected each animal with 10 mL of the inoculum containing 8.4 × 10^5^ EqHV RNA copies per mL. All animals became RNA-positive within three days and showed comparable EqHV RNA peak levels with titers reaching up to 10^6^ genome equivalents per mL serum (Fig. 1B). Furthermore, RNA-levels declined when the animals seroconverted and produced anti-NS3 antibodies. Notably, the horse seroconverted 35 days post-infection, while the donkeys seroconverted at day 42 and 48. Additionally, seroconversion was also associated with an increase of plasma baseline levels of GLDH and GGT liver enzymes in all three animals, while less pronounced in the donkeys (Fig. 1C). AST levels as well as fibrinogen were only elevated in the horse. The total protein levels (TP) as well as white blood cell counts (WBC) remained stable in all animals. Notably, reference interval data for the donkeys was taken from horses.

**Fig. 2:**
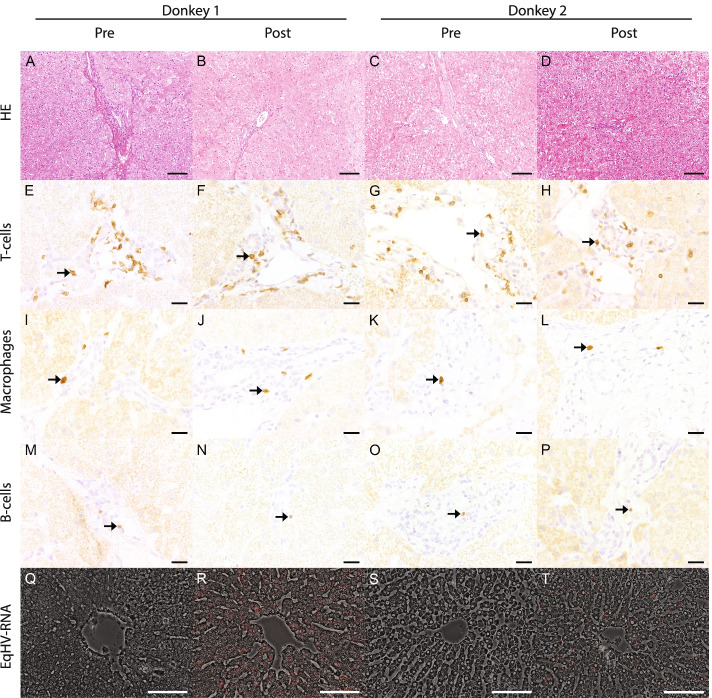
Morphological findings in liver biopsies from donkeys pre- and post- infection with EqHV. **A-D** Histologically, a mild periportally-accentuated, lympho-histiocytic inflammation was detected within liver biopsies with a similar appearance in samples taken pre- (**A, C**) and post- (**B, D**) infection. Hematoxylin and eosin, scale bar = 100 μm. **E-P** Inflammatory cell infiltrates consisted mainly of T-lymphocytes (**E-H**, asterisk), fewer macrophages (**I-L**, asterisk) and single B-lymphocytes (**M-P**, asterisk) pre- and post-infection. Immunohistochemistry, scale bar = 100 μm. **Q-T** Fluorescence in situ-hybridization lacked a positive reaction pre-infection (**Q, S**). Post-infection, EqHV specific RNA sequences were detected within the cytoplasm (**R, T**). Scale bar = 100 μm

Histologically, a mild, multifocal, periportally accentuated lympho-histiocytic hepatitis with few eosinophils was found in both donkeys (Fig. 2). Furthermore, single degenerated or necrotic hepatocytes partially accompanied by few neutrophils were present in donkey 1. Histopathological findings did not differ significantly pre- and post-infection. Periportal inflammatory cell infiltrates consisted mainly of T lymphocytes (94.47–97.86%), fewer macrophages (1.32–5.26%) and single B lymphocytes (0.26–0.81%) with similar values pre- and post-infection. FISH revealed EqHV-specific RNA sequences in 74.01 and 87.78% of hepatocytes after infection, in donkey 1 and donkey 2 respectively (Fig. 2). Samples obtained prior to infection lacked a specific signal by FISH. In conclusion, EqHV in donkey’s follows a similar infection kinetic, including viral load and antibody response. Moreover, liver enzymes increased slightly around the time of seroconversion in the absence of pathological findings.

### Individual transcriptomic changes post-infection

We recently showed that EqHV seroprevalence was comparable between donkey and horse cohorts, but RNA-prevalence was much lower in donkeys [7], indicating that donkeys might have a higher capacity to clear the infection. Therefore, to analyze immune patterns and individual responses to the EqHV infection, we characterized changes in the transcriptome in liver biopsies pre- and post-inoculation. Post-infection liver biopsies were taken at day 9, 10, and 7 for Donkey 1, Donkey 2 and the horse, respectively (Fig. 1). Each sample was sequenced twice on different lanes with high similarity between the four replicates (Fig. S2). All samples showed high expression of liver markers, while lung markers were not enriched (Fig. S2A). Furthermore, the principal component analysis (PCA) confirmed high similarity between replicates and indicated a stronger response to the virus infection in donkey 2 and the horse than in donkey 1 (Fig. S2B).

**Fig. 3:**
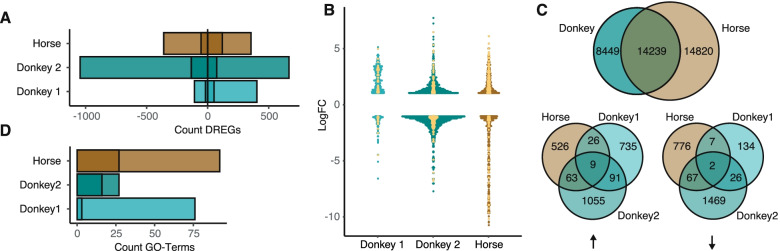
Analysis of deregulated genes. The threshold for deregulated genes (DREG) was set at a fold change above or below 2 or − 2, a significant *p*-value (FDR < = 0.05) and reads per kilobase million mapped reads (RPKM) of at least 1. **A** Number of up- and down-regulated genes per sample. Saturated area corresponds to immune-associated genes. **B** Overview of DREGs within each sample. Yellow dots indicate immune-associated genes. **C** Overlap of genes between the donkey and horse reference genome (upper Venn diagram). Overlap of all up- and down-regulated genes (indicated by arrows)

Next, we analyzed deregulated genes (DREGs), defined as fold change above 2 or below − 2, *p*-value < 0.05 and RPKM above 1. The number of DREGs was highest in donkey 2 (up: 666, down: 1045) followed by the horse (up: 360, down 355). For both animals, a small fraction was accounted by interferon regulated genes (IRGs), pointing towards an infection mediated response (Fig. 3A). Notably, donkey 1 showed a few down-regulated genes (108) while having more up-regulated genes (403) and only a few IRGs were deregulated (Fig. 3 A). Similarly, the genes with the highest fold change were present in the horse or donkey 2 transcriptome, while fold changes remained modest for donkey 1 (Fig. 3B). Subsequently, we characterized genes that were uniformly deregulated across all animals. This, however, was restricted since the reference genomes were not well annotated for both species, leaving a maximum overlap of 14,239 genes between the donkey and horse genome (14,820 unique horse genes, 8449 unique donkey genes). The overlap of up- and down-regulated genes remained modest, with only nine up- and two down-regulated genes shared between the animals (Fig. 3C, Fig. S3). Due to the close phylogenetic relatedness of EqHV to HCV, we evaluated the expression levels of HCV-related entry factors which were expressed on a high level in all animals (Fig. S4). Notably, it is yet unclear which receptors are being used by EqHV, or other non-human hepaciviruses, to enter their host cells.

To classify gene expression patterns, we used a Gene Ontology (GO) analysis which showed multiple terms activated in the horse (92) and donkey 1 (76), while only a modest number of terms was enriched in donkey 2 (27). We highlighted terms associated with immune defense mechanisms, showing that the horse had the largest number of them being activated, followed by donkey 2 and donkey 1 (Fig. 3D). Altogether, the three animals showed a clear change in their transcriptome when comparing the pre- to the post-infection liver biopsies. Moreover, the response was highly individual, and the horse and donkey 2 had a stronger induction of immune associated genes than donkey 1.

**Fig. 4:**
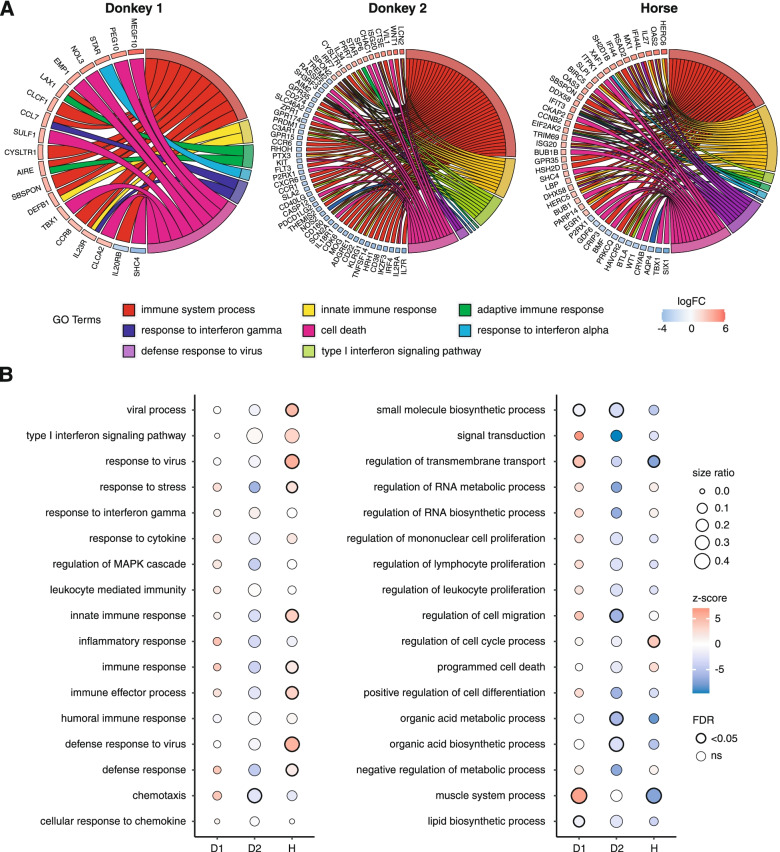
Analysis of immune signatures in donkeys and the horse. **A** Chord plots depicting the 250 most de-regulated genes for each animal and their contribution to the (de-)activation of immune system processes. **B** Regulation of selected GO-terms. The dot color reflects whether a term is up- or down-regulated (z-score). The size ratio indicates the number of deregulated genes per term and the dot border is a binary indicator for a significant (FDR < = 0.05) deregulation

To evaluate which arm of the immune system was activated, we plotted the 250 most up- and down-regulated genes that cluster in either one of the following GO-terms: Immune system process, innate immunity, adaptive immune response, response to interferon alpha, response to interferon gamma, defense response to virus or cell death (Fig. 4A). This analysis suggests that the immune response in donkey 1 was weaker and less virus-specific than in donkey 2 and the horse. Both animals had a strong activation of immune system process associated genes and genes belonging to the adaptive immunity. However, there were marked differences within the virus-specific defense which was more pronounced in the horse. Moreover, the genes that cluster in those pathways were mostly up-regulated within the horse transcriptome, while in contrast, they were mostly down-regulated for the donkey 2 transcriptome.

Additionally, we were not able to identify immune ontologies that were similarly deregulated (Fig. 4B). Most strikingly, the GO-terms viral process, response to virus, innate immune response, and defense response to virus were all significantly activated in the horse, and in contrary to this, down-regulated in the donkeys. Only a few terms were similarly expressed, but were not directly linked to immune processes, e.g. the term small molecule biosynthetic process.

In conclusion, the expression of immune-associated genes was more pronounced in the horse and donkey 2 compared to donkey 1. Moreover, specific anti-viral expression was only apparent in the horse.

### Disease ontology classification shows association with viral hepatitis in horses but not in donkeys

**Fig. 5:**
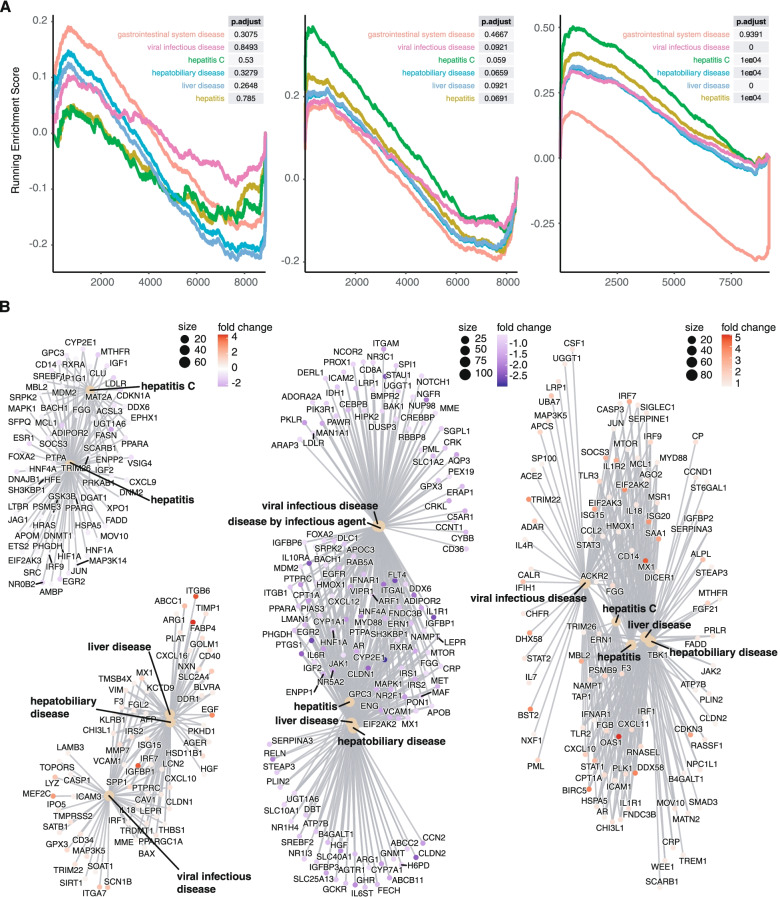
Disease ontology classification. **A** Gene set enrichment analysis (GSEA) for terms associated with viral liver hepatitis. **B** Network analysis of the same terms showing the interaction between terms and involved genes

To assess whether transcriptomic responses were associated with signs of liver disease or viral infections, we deployed disease ontology classification. We first calculated the gene set enrichment (GSEA) score for the following terms: Disease by infectious agents, gastrointestinal system disease, hepatitis, hepatitis C, hepatobiliary disease, liver disease and viral infectious disease (Fig. 5A). We chose those terms because (i) EqHV is an infectious agent, possibly connected to an anti-viral response and (ii) hepaciviruses are hepatotropic and thus likely causing liver damage such as hepatitis. The enrichment score analysis showed a significant enrichment for hepatitis, hepatitis C, hepatobiliary disease, liver disease and viral infectious disease in the horse, while donkey 2 only had a significant enrichment for the hepatitis C term and donkey 1 showed no significantly enriched terms. However, in both donkeys a non-significant enrichment for liver disease and hepatitis was present. Subsequently, we visualized how those terms were linked to one another, which showed that for the horse there was a strong connection between liver pathology and viral infection, most likely driven by the activation of ISGs such as OAS1, MX1, and ISG20 (Fig. 5B). There was no apparent connection between the disease terms for donkey 1, and only a weak one for donkey 2 which involved ISGs like ISG15, ISG20, and LCN2.

In concordance with the previous analysis, virus specific immune response and liver inflammation was only apparent in the horse. Donkey 2 showed a weak induction of hepatitis related terms, while donkey 1 did not show any enrichments.

## Discussion

The genus of hepacivirus comprises multiple viruses each infecting their individual host [1]. Cross-species transmission is rarely reported in this genus and only experimental transmission of the human pathogen the hepatitis C virus into chimpanzees has been shown. In 2017, donkeys were shown to be infected with the equine hepacivirus, with a similar seroprevalence, but a lower rate of RNA-positive animals [7]. To characterize to which extent donkeys are susceptible and whether donkeys might be more resistant to EqHV than horses, we inoculated two donkeys and one horse which allowed us to analyze the course of disease and transcriptomic responses to the EqHV infection in liver biopsies.

After successfully infecting two donkeys and one horse with ultracentrifugation purified EqHV-positive serum, all animals showed a similar course of disease, including high viremia, delayed seroconversion. Liver enzymes serum levels were slightly elevated in the horse at the time of seroconversion, which was less pronounced in the donkeys. Furthermore, the RNA titer decreased with increasing anti-NS3 antibody titer, indicating that the adaptive immunity plays an important role in clearing the infection. However, in the horse EqHV-RNA quantities quickly increased back to previous levels, suggesting that this animal might transit into a persistent infection. Unfortunately, we were not able to monitor this horse longer than 56 days, leaving uncertainty about whether it became persistently infected. In previous studies, horses were infected with non-purified EqHV-positive serum, which could have led to the stimulation of the recipient’s immune system and consequently contributing to viral clearance [8, 9]. Hence, future studies could access the influence of the infection, namely inoculum viral load and adjuvants, method on viral kinetics. Overall, EqHV kinetics are in line with previous studies that showed high RNA titers, delayed seroconversion followed by liver enzyme elevation in horses and similarly in HCV infected chimpanzees and humans [3, 8, 9, 25–27].

The low rate of RNA-positive donkeys in previously screened cohorts suggested either inefficient replication or higher resistance in donkeys. The inability of efficient propagation of a virus which primarily infects horses in donkeys has been shown for the Equine Infectious Anemia Virus (EIAV), where duration of infection, viral loads and clinical symptoms were less pronounced in donkeys than in horses [28]. Similarly, donkeys are more resistant towards African horse sickness (AHS), West Nile fever (WNF) and equine viral arteritis (EVA) (reviewed in [20]). In this study, high titer replication and long-term infection of EqHV in donkeys were similar to that in the horse, pointing towards higher resistance in donkeys, possibly mediated by differences in immune responses. Interestingly, within the genus Equidae which includes wild and domesticated species, immune genes were among a set of genes under strong positive selection during equine evolution [18].

We subsequently analyzed the transcriptomic response in pre- and post-infection liver biopsies. Distinct patterns for each animal were detected instead of a uniform set of genes associated with disease or clearance. High karyotypic heterogeneity and substantial heterogeneity of copy numbers between donkeys and horses might have contributed to the high variation [29, 30], and additionally, the not necessarily well annotation reference genomes for the horse (EquCap3.0) and donkey (ASM303372v1) hindered detailed analysis. Notably, 15.4% of the horse genome and 10.4% of the donkey genome were not identified within the respective opposite genome [31]. Likewise, the individual genetic background, age, breed, and fitness could have played an important role.

Overall, we were able to show that immune responses, especially towards viral defense, were more pronounced in the horse than in the donkeys. Notably, donkey 1 showed only a weak response in general, while donkey 2 mounted a stronger immune response. Interestingly, even though donkey 1 had a weak immune response and the slowest seroconversion, it was able to clear the infection within the same timeframe as donkey 2. This might indicate that other factors which were not activated at this early state within the liver, such as the adaptive immunity, were strongly involved in viral clearance. It has already been shown that HCV-clearance is associated with a strong response of CD4 and CD8 T-cells in humans and chimpanzees [32]. Likewise, a moderate activation of EqHV-specific CD4 and CD8 T-cells was shown in acutely infected horses. However, this process in horses is not entirely understood yet [21]. Interestingly, modern horses were found to have a significant enrichment of immune-related genes, potentially contributing to the stronger immune response in the horse than in the donkeys [15].

The evolutionary origin of the hepatitis C virus remains unknown. Model systems, such as the EqHV infections in equids, could help understand viral and host determinants required for species barrier crossing. For instance, recently, a study about intra-host diversity of EqHV and HCV shed light into viral determinants important for persistent hepacivirus infection and indicated the hypervariable region 1 in HCV as an important determinant in infecting the human population [33]. Furthermore, a previous study by Hoffmann et al. 2020 found a divergent EqHV sequence in donkeys that differed by 22% from the reference strain which could have an impact on EqHV diagnostics in donkeys [34]. Thus, characterizing host mediated immune pressure on the virus population could help to understand important factors in establishing infections while crossing the natural species barrier. Similarly, donkey – horse infections are being used to elucidate determinants of species barriers for the EIA virus [20].

The clinical sings of EqHV in horses or HCV in humans are usually absent or moderate, especially during the early stage of infection [35]. This is in line with little pathological alterations within liver biopsies from day 7–9. However, disease ontology analysis allowed us to analyze gene expression patterns that correlate with signs of liver inflammation or viral infection at this early stage. The horse showed the most specific pattern for a viral infection and hepatitis. Donkey 2 showed a weak response towards viral infection, including hepatitis, while donkey 1 showed a non-specific response. Overall, this pattern fits well to the transcription analysis.

This study clearly has its limitations. As this was a proof of principle study, we were only able to infect a limited number of animals for a limited time span. Therefore, we were not able to find a common pattern during EqHV infection or clearance. Furthermore, we were not able to investigate longitudinal responses or long-term effects of EqHV on the immune system. On the other hand, we used a novel inoculation method to show that donkeys are susceptible to EqHV infection and follow a similar course of disease, which includes comparable serum EqHV RNA titers, delayed seroconversion, and slight elevation of liver enzymes during seroconversion, suggesting that the donkey is also a natural host for EqHV infections. We additionally analyzed the transcriptomic response between pre- and post-infection of liver biopsies, which showed individual expression pattern for each animal and stronger activation of the immune system in the horse than in the donkeys.

## Conclusion

In this study we were able to show that EqHV infects both donkeys and horses in a similar fashion. The course of EqHV-RNA levels was almost identical, suggesting that donkeys might as well be a natural host of this virus. However, immune mechanisms in response to the virus infection seems to differ between the animals.

## Supplementary Information

**Additional file 1.**

## Data Availability

All data will be made available upon request.
