# Supplementary material for: Experimental cross-species infection of donkeys with equine hepacivirus and analysis of host immune signatures
Source: One Health Outlook. 2022 May 9;4:9. doi: 10.1186/s42522-022-00065-y (PMC9082851; doi:10.1186/s42522-022-00065-y)
Supplement: Supplementary file 1 — Additional file 1. [file 42522_2022_65_MOESM1_ESM.pdf]

Supplementary Information to:

Experimental cross-species infection of donkeys with Equine Hepacivirus and analysis of host immune signatures

André Gömer[1,2], Christina Puff[3], Birthe Reinecke[4], Stephanie Bracht[4], Maria Conze[5], Wolfgang Baumgärtner[3], Jörg Steinmann[6, 7], Karsten Feige[5], Jessika M.V. Cavalleri[8], Eike Steinmann[1], Daniel Todt[1,9*]

[1]Department of Molecular and Medical Virology, Ruhr University Bochum, Bochum, Germany
[2]Institute of Virology, University of Veterinary Medicine Hannover, Foundation, Hannover, Germany
[3]Department of Pathology, University of Veterinary Medicine Hanover, Foundation, Hannover, Germany
[4]Institute of Experimental Virology, TWINCORE Center for Experimental and Clinical Infection Research, Hannover, Germany
[5]Clinic for Horses, University of Veterinary Medicine Hannover, Foundation, Hannover, Germany
[6]Institute of Medical Microbiology, University of Hospital Essen, University of Duisburg-Essen, Essen, Germany
[7]Institute of Clinical Hygiene, Medical Microbiology and Infectiology, General Hospital Nürnberg, Paracelsus Medical University, Nürnberg, Germany.
[8]Clinical Department for Internal Medicine for Horses, Veterinary University of Vienna, Vienna, Austria
[9]European Virus Bioinformatics Center (EVBC), Jena, Germany

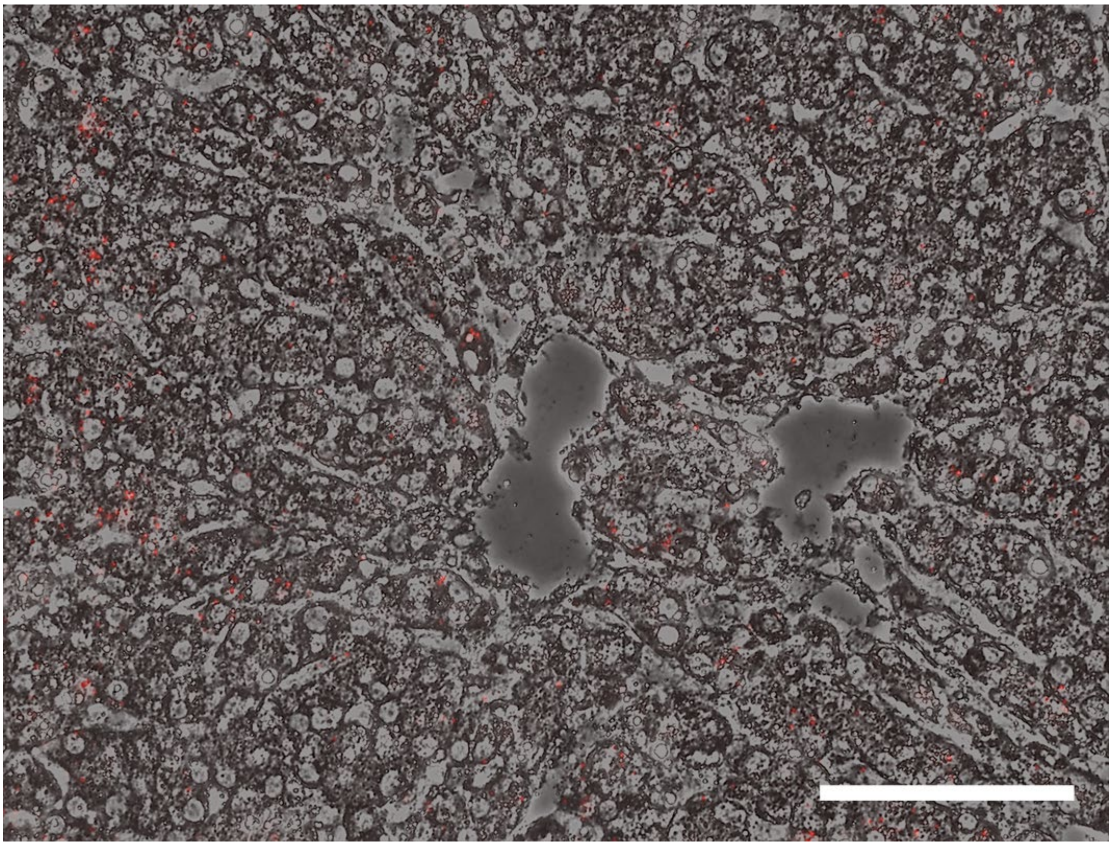

Figure S1: Fluorescence in situ-hybridization positive control. EqHV specific RNA sequences were detected within the cytoplasm (red dots). Positive control used in the assay for Figure 2. Scale bar = 100 µm.

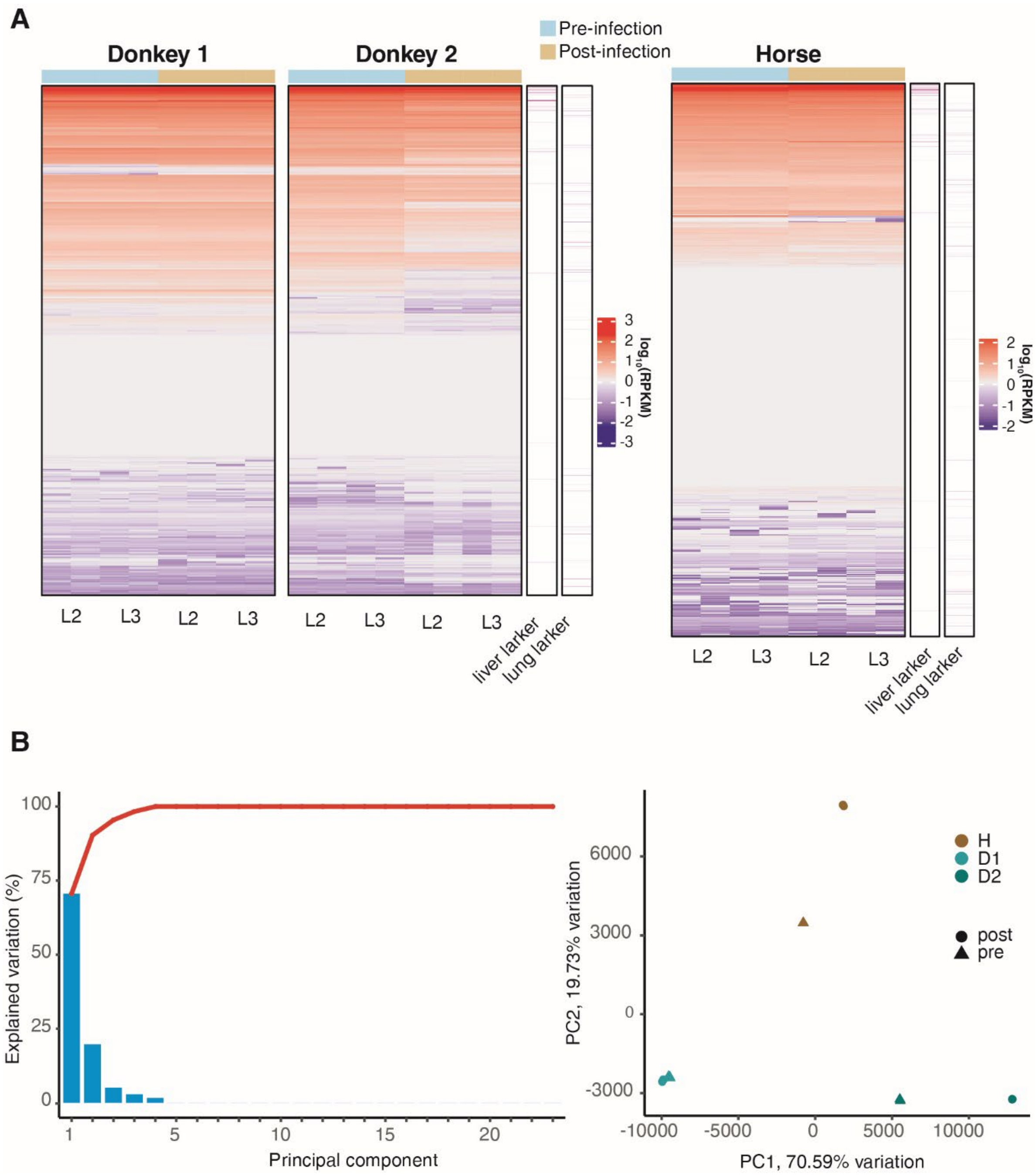

Figure S2 NGS data quality control. (**A**) Overview of all genes identified in sequencing runs per paired-end read and lane (L2, L3). In total, each sample was sequenced four times. Liver-specific marker genes accumulate among the highly expressed transcripts, while lung-specific markers are randomly distributed in the clustered heat map. (**B**) Principle Component Analysis (PCA) of expression profiles for each donkey and horse pre- and post-infection. Two-dimensional representation of the two principle components (PC1 and PC2) with the highest expected information yield (explained variation %).

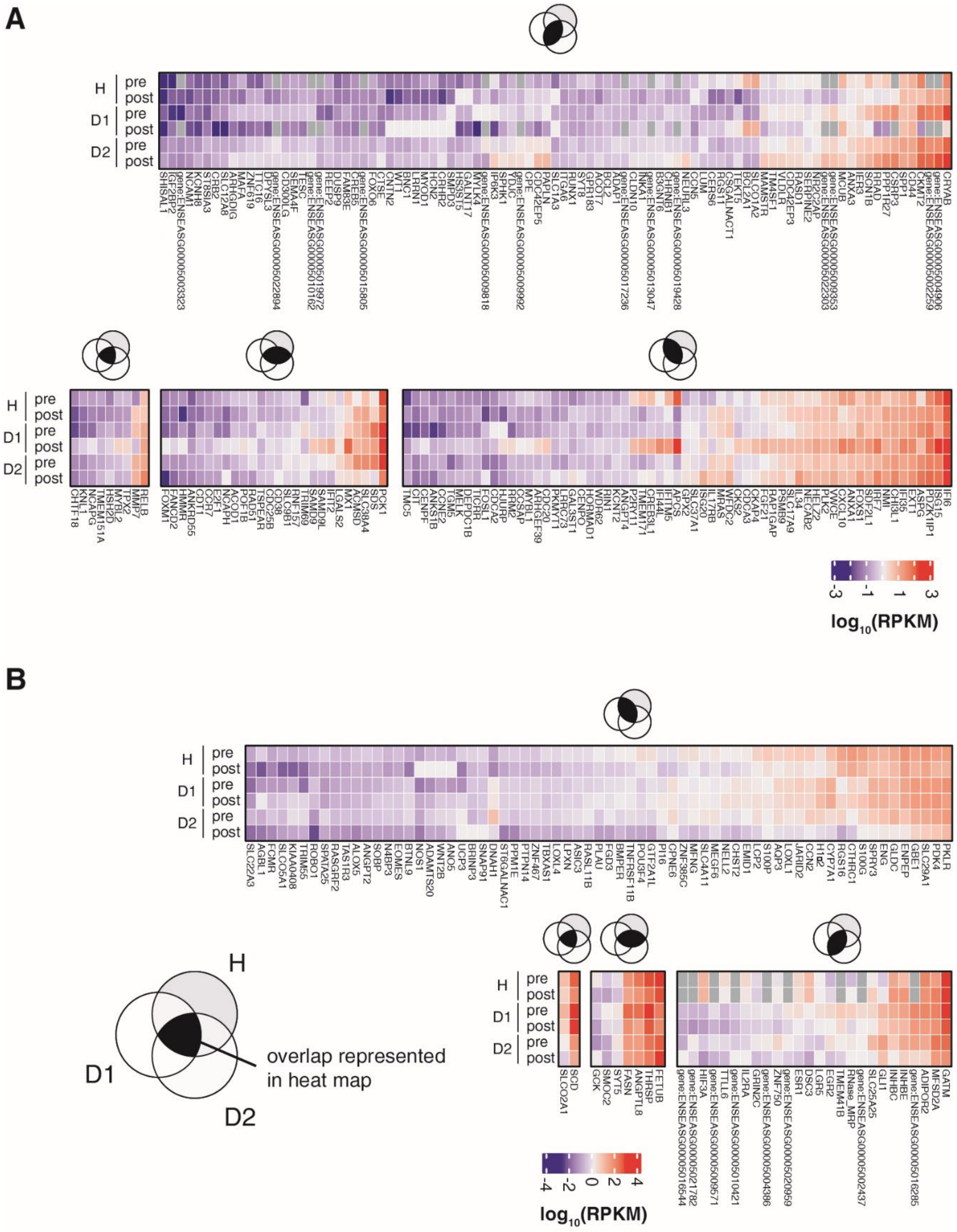

Figure S3: Mutually deregulated genes. Expression values of genes that were exclusively up (**A**)- or down- regulated (**B**) between pre- and post-infection liver samples and shared between indicated datasets (Venn diagrams).

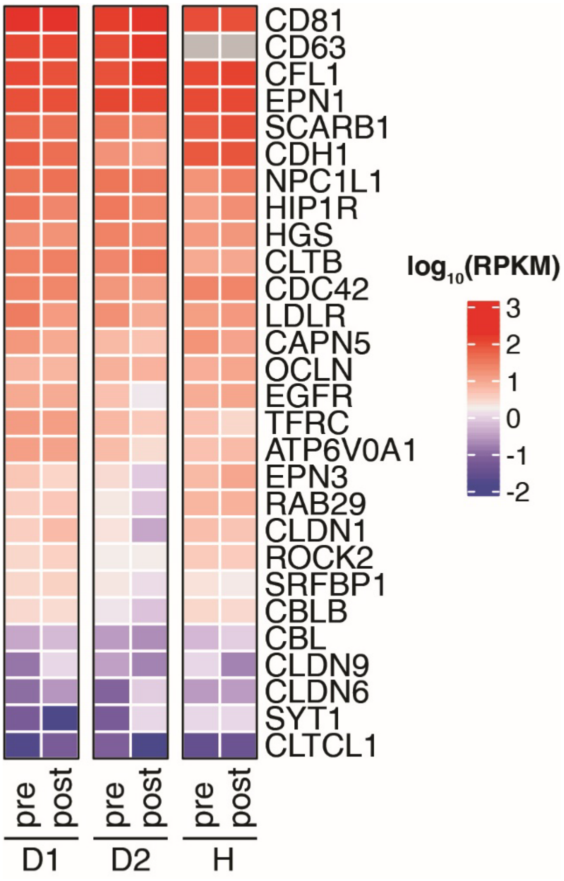

Figure S4: Potential entry factors. Expression patterns pre- and post-infection in the two donkeys and the control horse of factors described in the context of HCV entry in humans.
